# Public health indicators for the EU: the joint action for ECHIM (European Community Health Indicators & Monitoring)

**DOI:** 10.1186/0778-7367-71-12

**Published:** 2013-05-30

**Authors:** Marieke Verschuuren, Mika Gissler, Katri Kilpeläinen, Antti Tuomi-Nikula, Ari-Pekka Sihvonen, Jürgen Thelen, Rita Gaidelyte, Silvia Ghirini, Nils Kirsch, Remigijus Prochorskas, Emanuele Scafato, Pieter Kramers, Arpo Aromaa

**Affiliations:** 1 RIVM, National Institute for Public Health and the Environment, P.O. Box 1, Bilthoven, BA, NL - 3720, the Netherlands; 2 THL, National Institute for Health and Welfare, P.O. Box 30, Helsinki, FI - 00270, Finland; 3 Robert Koch Institute, P.O. Box 65 02 61, D-13302, Berlin, Germany; 4 Center of Health Information, Institute of Hygiene, Didzioji 22, Vilnius, LT – 01128, Lithuania; 5 ISS, Istituto Superiore di Sanità, Viale Regina Elena, 299, Rome, IT - 00161, Italy; 6 Ratakatu 1a, Helsinki, FI-00120, Finland

**Keywords:** Public health indicators, Public health monitoring, Public health reporting

## Abstract

**Background:**

Public health policies aim to improve and maintain the health of citizens. Relevant data and indicators are needed for a health policy that is based on factual information. After 14 years of work (1998–2012), the multi-phase action on European Community Health Indicators (ECHI) has created a health monitoring and reporting system. It has generated EU added value by defining the ECHI shortlist with 88 common and comparable key health indicators for Europe.

**Methods:**

In the 2009-2012 Joint Action for ECHIM project the ECHI shortlist was updated through consultation with Member State representatives. Guidelines for implementation of the ECHI Indicators at national level were developed and a pilot data collection was carried out.

**Results:**

67 of the ECHI Indicators are already part of regular international data collections and thus available for a majority of Member States, 14 are close to ready and 13 still need development work. By mid-2012 half of the countries have incorporated ECHI indicators in their national health information systems and the process is ongoing in the majority of the countries. Twenty-five countries were able to provide data in a Pilot Data Collection for 20 ECHI Indicators that were not yet (fully) available in the international databases.

**Conclusions:**

The EU needs a permanent health monitoring and reporting system. The Joint Action for ECHIM has set an example for the implementation of a system that can develop and maintain the ECHI indicators,, and promote and encourage the use of ECHI in health reporting and health policy making. The aim for sustainable public health monitoring is also supported by a Eurostat regulation on public health statistics requiring that health statistics shall be provided according to the ECHI methodology. Further efforts at DG SANCO and Eurostat are needed towards a permanent health monitoring system.

## Background

The major aim of public health policies is to improve and maintain the health of citizens and to reduce health inequalities. These policies have to be based on factual information drawn from relevant data and comparable indicators. Relevant health indicators enable correctly targeted policy measures and assessment of their impact.

To reach this goal, health indicators have to be based on representative population-based health data and need to be comparable between points in time, countries and areas. Observed differences between countries can stimulate the improvement of national health systems. Therefore, a joint international health information system will help Member States to implement their public health monitoring and reporting system and thus enable them to carry out their public health responsibilities. For the EU, the implementation of relevant health indicators is an essential starting point for a common health monitoring and reporting system that is essential for supporting EU level public health policies.

The European Parliament has called for an effective health information system since the 1990s. The first step on the road to harmonisation was the launch of the European Commission’s first Health Monitoring Programme in 1993. Under this Programme projects were financed to develop harmonized health indicators [1]. In 1996, the European Commission set up a working group to draft a proposal on how to organise health monitoring in the European Union [2]. The following year, the Amsterdam Treaty provided harmonised instructions on the public health responsibilities of the Member States [3].

The multi-phase action on European Community Health Indicators (ECHI) has been one of the core actions of the European Commission’s Health Programmes for 14 years (1998–2012). Its main task was the development, maintenance and implementation of a set of general public health indicators, the ECHI shortlist. Several international indicator- and datasets already exist, both broad (e.g. Eurostat, WHO Health for All database, OECD health data) and topical (e.g. data collections by the EMCDDA and ECDC). Yet the ECHI shortlist provides added value because it has been developed as a concise yet comprehensive tool for policy support, rather than as a (data driven) database. The first two projects (ECHI 1998–2001 and ECHI-2 2002–2004) focused on the selection and definition of indicators, and established the first version of the ECHI shortlist in 2005 [4,5]. The 3^rd^ (ECHIM 2005–2008, M stands for Monitoring) [6] and the 4^th^ phase (Joint Action for ECHIM 2009–2012) shifted the focus towards the implementation of the ECHI indicators in the Member States and at EU level. A Joint Action is a specific financing mechanism that was newly introduced together with the EU Health Programme Together for Health in 2008. It involves a closed call from the Commission to the Member States to present a proposal, in contrast with normal project calls, which are open. In 2012, Prof. Aromaa described his personal reflections on the progress of the ECHI(M) projects in the broader perspective of past as well as necessary future developments [7].

The incorporation of ECHI Indicators into national health information systems is essential to ensure the continuous development and improvements in ECHI data availability, quality and comparability in the EU. During the 3^rd^ project phase (2005–2008) a start has been made with this implementation of the ECHI Indicators at national level by, among other things, setting up a network of ECHI contact persons in the EU Member States and assessing availability of national data for ECHI Indicators in international databases [6]. The Joint Action for ECHIM continued this implementation work. Next to enhancing implementation of ECHI Indicators at national level, its main objectives comprised updating and documenting the ECHI shortlist, and the assessment of availability and quality of data for ECHI Indicators that are not yet part of existing international data collections by means of a data collection pilot. In this article, we will describe the main results and experiences of the Joint Action for ECHIM.

## Methods

There were five project partners in the Joint Action: the public health institutes of Finland (main partner), the Netherlands, Germany, Lithuania and Italy. Twenty-four Member States in total gave an official declaration of intent to participate in the Joint Action [8]. In practice, though, 36 countries (EU Member States, accession and candidate countries, and EFTA countries) participated. The Joint Action started on 1 January 2009 and ended on June 30^th^ 2012. Further methods applied are described according to the following three main objectives of the Joint Action:

### An updated and fully documented shortlist of ECHI indicators

A new procedure for updating the shortlist was developed in 2010–2011, together with the Member State representatives of all countries participating in the Joint Action. Application of this new procedure resulted in the 2012 version of the ECHI shortlist. Clear criteria for additions or removals of indicators to/from the shortlist are at the core of the new updating procedure. Furthermore, the strong focus of the Joint Action on implementing the indicators is reflected in the criteria as well. The updating procedure has been described in detail in the final report of the Joint Action part II [9].

### Implementation of the ECHI shortlist indicators in participating EU countries

The project partners created a model for the implementation plans for ECHI indicators, consisting of several elements (e.g. communication, data availability). Based on this model, guidelines for the Member States were developed at the beginning of the project. Progress of national implementation was monitored. The guidelines are described in detail in the Joint Action final report part I [10].

### Pilot data collection

The existing international databases of Eurostat, the WHO Health for All database and OECD Health Data together with topic-specific international databases (e.g. ECDC and EMCDDA) are the recommended data source for 44 shortlist indicators. An ECHIM Pilot Data Collection was performed in 2010–2011 to obtain comparable data for 20 ECHI shortlist indicators that were unavailable or incomparable in these international databases. For many of these 20 indicators the European Health Interview Survey (EHIS) is the preferred data source. Therefore, ECHI-conform data were obtained from Eurostat for Member States for which EHIS micro-datasets were available from EHIS wave I, which was carried out in the period 2006–2010 [11]. These were complemented with data from national Health Interview Surveys from Member States that had not participated in EHIS wave I. Out of the 36 countries participating in the Joint Action, in 34 suitable contact persons were identified for receiving a Pilot Data Collection questionnaire.

## Results

### An updated and fully documented shortlist of ECHI indicators

After introducing the ECHI shortlist in 2005 [5], the indicator metadata for all 88 indicators in the ECHI shortlist has been documented and continuously improved, and the ECHI shortlist has been updated in 2008 [6] and 2012 [9]. The 2008 version comprised an implementation and a development section. During the Joint Action, however, a more precise definition of the indicators and a stronger focus on implementation led to splitting the development section into a work-in-progress section in addition to the development section. Therefore, the 2012 version of the ECHI shortlist is divided into three rather than two sections.

The 67 ECHI Indicators in the implementation section are already part of regular international data collections, and data are available for a majority of Member States, and thus ready for implementation. The 14 ECHI indicators in the work-in-progress section are almost ready to be included in regular international data collections. In most cases, however, no concrete plans exist for this at present. The remaining 13 ECHI indicators in the development section contain topics that are needed for policy support, but that are not ready yet for incorporation in international regular data collections and for implementation. Please note that there are 88 indicators in the ECHI shortlist. However, six of these have two different operationalizations: one based on self-reported data and one based on administrative or register-based data. Both operationalizations have been assessed separately here, resulting in a total of 67 + 14 + 13 = 94 indicators. An overview of the 2012 version of the ECHI shortlist is presented in Table 1.

**Table 1:** ECHI shortlist, 2012 version

**ECHI shortlist indicators**	**Data source**	**Status indicator**
1. Population by sex/age	Eurostat	Implementation section
2. Birth rate, crude	Eurostat	Implementation section
3. Mother’s age distribution	Eurostat	Implementation section
4. Total fertility rate	Eurostat	Implementation section
5. Population projections	Eurostat	Implementation section
6. Population by education	Eurostat (LFS)	Implementation section
7. Population by occupation	Eurostat (LFS)	Implementation section
8. Total unemployment	Eurostat (LFS)	Implementation section
9. Population below poverty line and income inequality	Eurostat (EU-SILC)	Implementation section
10. Life expectancy	Eurostat	Implementation section
11. Infant mortality	Eurostat	Implementation section
12. Perinatal mortality	WHO-HFA	Implementation section
13. Disease-specific mortality	Eurostat (and CISID for AIDS-related mortality)	Implementation section
14. Drug-related deaths	EMCDDA	Implementation section
15. Smoking-related deaths	n.a.	Work-in-progress section
16. Alcohol-related deaths	n.a.	Work-in-progress section
17. Excess mortality by extreme temperatures	n.a.	Development section
18. Selected communicable diseases	ECDC	Implementation section
19. HIV/AIDS	EURO-HIV/CISID	Implementation section
20. Cancer incidence	Globocan	Implementation section
21. (A) Diabetes, self-reported prevalence	Eurostat (EHIS)	Implementation section
21. (B) Diabetes, register-based prevalence	n.a.	Work-in-progress section
22. Dementia	n.a.	Work-in-progress section
23. (A) Depression, self-reported prevalence	Eurostat (EHIS)	Implementation section
23. (B) Depression, register-based prevalence	n.a.	Work-in-progress section
24. Acute Myocardial Infarction	n.a.	Work-in-progress section
25. Stroke	n.a.	Work-in-progress section
26. (A) Asthma, self-reported prevalence	Eurostat (EHIS)	Implementation section
26. (B) Asthma, register-based prevalence	n.a.	Work-in-progress section
27. (A) COPD, self-reported prevalence	Eurostat (EHIS)	Implementation section
27. (B) COPD, register-based prevalence	n.a.	Work-in-progress section
28. (Low) birth weight	WHO-HFA	Implementation section
29. (A) Injuries: home/leisure, self-reported incidence	Eurostat (EHIS)	Implementation section
29. (B) Injuries: home/leisure, register-based incidence	IDB	Implementation section
30. (A) Injuries: road traffic, self-reported incidence	Eurostat (EHIS)	Implementation section
30. (B) Injuries: road traffic, register-based incidence	UN ECE	Implementation section
31. Injuries: workplace	Eurostat (ESAW)	Implementation section
32. Suicide attempt	n.a.	Development section
33. Self-perceived health	Eurostat (EU-SILC)	Implementation section
34. Self-reported chronic morbidity	Eurostat (EU-SILC)	Implementation section
35. Long-term activity limitations	Eurostat (EU-SILC)	Implementation section
36. Physical and sensory functional limitations	Eurostat (EHIS)	Implementation section
37. General musculoskeletal pain	n.a.	Development section
38. Psychological distress	n.a.	Development section
39. Psychological well-being	n.a.	Development section
40. Health expectancy: Healthy Life Years (HLY)	Eurostat	Implementation section
41. Health expectancy, others	EHEMU/EHLEIS project	Work-in-progress section
42. Body mass index	Eurostat (EHIS)	Implementation section
43. Blood pressure	Eurostat (EHIS)	Implementation section
44. Regular smokers	Eurostat (EHIS)	Implementation section
45. Pregnant women smoking	n.a.	Work-in-progress section
46. Total alcohol consumption	WHO (GISAH)	Implementation section
47. Hazardous alcohol consumption	Eurostat (EHIS)	Implementation section
48. Use of illicit drugs	EMCDDA	Implementation section
49. Consumption of fruit	Eurostat (EHIS)	Implementation section
50. Consumption of vegetables	Eurostat (EHIS)	Implementation section
51. Breastfeeding	WHO-HFA	Work-in-progress section
52. Physical activity	Eurostat (EHIS)	Implementation section
53. Work-related health risks	EUROFOUND	Implementation section
54. Social support	Eurostat (EHIS)	Implementation section
55. PM10 (particulate matter) exposure	Eurostat	Implementation section
56. Vaccination coverage in children	WHO-HFA	Implementation section
57. Influenza vaccination rate in elderly	Eurostat (EHIS)	Implementation section
58. Breast cancer screening	Eurostat (EHIS)	Implementation section
59. Cervical cancer screening	Eurostat (EHIS)	Implementation section
60. Colon cancer screening	Eurostat (EHIS)	Implementation section
61. Timing of first antenatal visits among pregnant women	n.a.	Work-in-progress section
62. Hospital beds	Eurostat	Implementation section
63. Practising physicians	Eurostat	Implementation section
64. Practising nurses	Eurostat	Implementation section
65. Mobility of professionals	n.a.	Development section
66. Medical technologies: MRI units and CT scans	Eurostat	Implementation section
67. Hospital in-patient discharges, limited diagnoses	Eurostat	Implementation section
68. Hospital day-cases, limited diagnoses	Eurostat	Implementation section
69. Hospital day-cases as percentage of total patient population (in-patients & day-cases), selected diagnoses	Eurostat (necessary discharge data available but ratio is not centrally computed yet)	Implementation section
70. Average length of stay (ALOS), limited diagnoses	Eurostat	Implementation section
71. General practitioner (GP) utilisation	Eurostat (EHIS)	Implementation section
72. Selected outpatient visits	Eurostat (EHIS)	Implementation section
73. Selected Surgeries	Eurostat	Implementation section
74. Medicine use, selected groups	Eurostat (EHIS)	Implementation section
75. Patient mobility	Eurostat is regularly collecting data on patient mobility but is not yet publishing these.	Work-in-progress section
76. Insurance coverage	OECD	Implementation section
77. Expenditures on health	Eurostat	Implementation section
78. Survival rates cancer	EUROCARE	Implementation section
79. 30-day in-hospital case-fatality Acute Myocardial Infarction and stroke	OECD	Implementation section
80. Equity of access to health care services	Eurostat (EU-SILC)	Implementation section
81. Waiting times for elective surgeries	n.a.	Development section
82. Surgical wound infections	n.a.	Development section
83. Cancer treatment delay	n.a.	Development section
84. Diabetes control	n.a.	Development section
85. Policies on ETS exposure (Environmental Tobacco Smoke)	WHO-Euro tobacco control (computation of indicator not done centrally yet)	Implementation section
86. Policies on healthy nutrition	n.a.	Development section
87. Policies and practices on healthy lifestyles	n.a.	Development section
88. Integrated programmes in settings, including workplace, schools, hospital	n.a.	Development section

A report documenting the indicators and the work performed has been published [9]. This ‘cookbook’ for the ECHI shortlist indicators is aimed at serving persons working with the indicators, computing them, or making them available. In addition, the European Commission made an up-to-date presentation of data and metadata for multiple indicators in its HEIDI tool [12].

### Implementation of the ECHI shortlist indicators in participating EU countries

During the Joint Action for ECHIM, ten countries already started incorporating ECHI into their national databases: Austria, Czech Republic, Estonia, Germany, Greece, Italy, Lithuania, Latvia, the Netherlands and Spain. Some countries have created national offline data presentations of ECHI indicators, for example using national customised versions of the WHO Data Presentation System (DPS), as has been done in Lithuania [13]. In addition, a number of other countries have started using the ECHI framework in their health reports, e.g. the Netherlands [14] and France [15]. A number of countries in June 2012 reported having concrete plans to incorporate ECHI in the national indicator and/or data presentation system (e.g. Malta, Finland, Ireland, and Norway). This shows for example from stating in future action plans of National Public Health Institutes or strategies of the Ministries of Health that there is an intention to use ECHI indicators in national health monitoring and reporting systems.

### Pilot data collection

**Figure 1:**
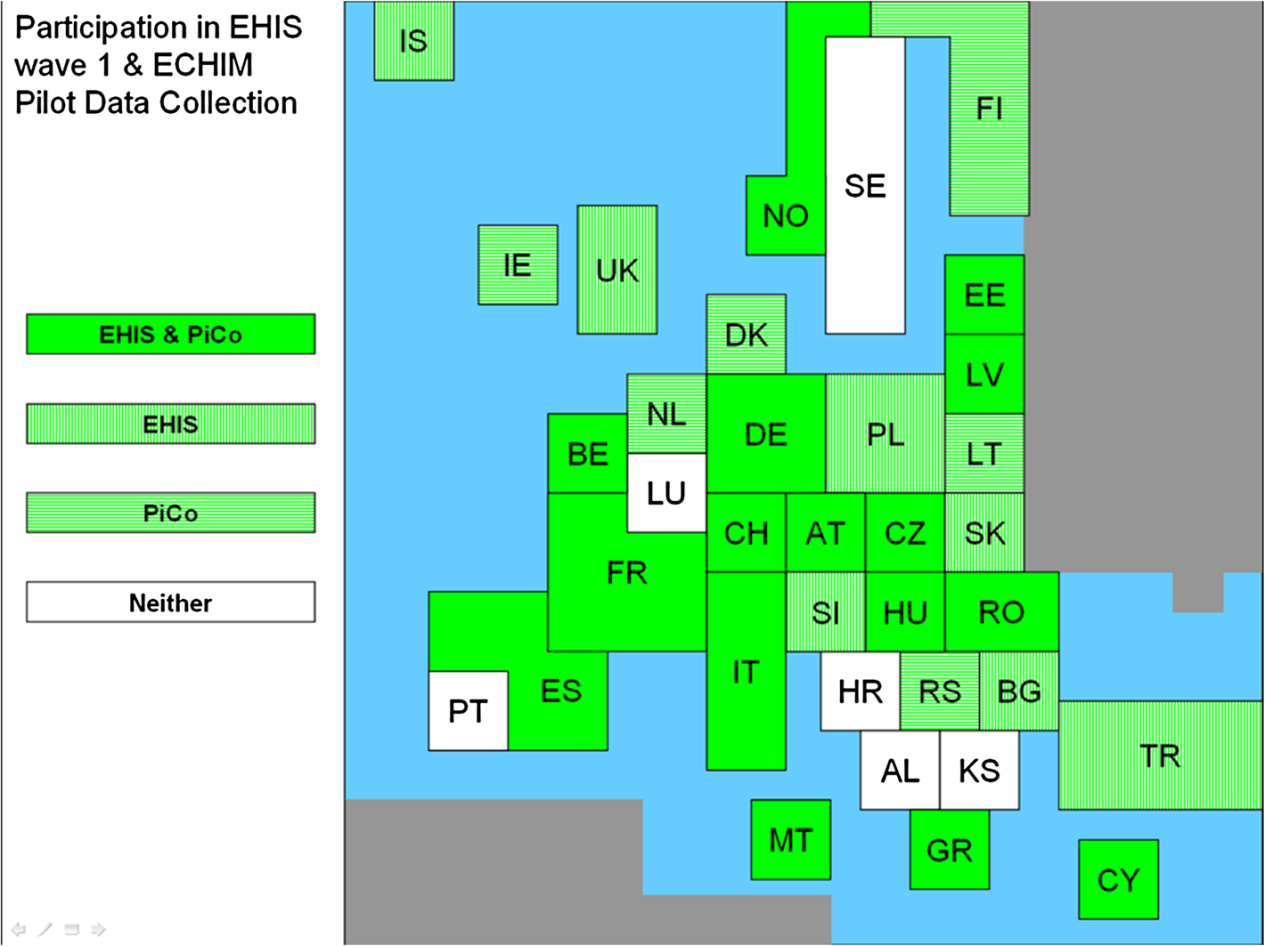
Participation in the ECHIM pilot data collection in 2011–2012 and wave I of European Health Interview Survey (EHIS) in 2006–2009.

In total 25 countries provided data in the pilot (see Figure 1). No pilot data at all were received from nine countries (Bulgaria, Greece, Luxembourg, Portugal, Sweden, Slovenia, Slovakia, Hungary and Turkey). However, none of the participating countries was able to provide all requested indicators and breakdowns. Furthermore, conceptual and methodological differences among national HIS data hampered valid mutual comparisons, as well as clear-cut comparisons with the EHIS-based data. A separate final report of the Joint Action documenting in detail the ECHIM Pilot Data Collection, data received and the analyses has been published in 2013 (part III) [16].

## Discussion

The European Parliament has highlighted the need for a public health information system. High quality health information serves the EU and Member States by helping to direct health, welfare and other policies and planning toward meeting peoples’ health needs. Comparative health information is of great practical use not only for policy makers and planners, but also for health professionals, teachers, students, researchers, journalists, and the general population.

There is ample evidence from many European countries about the usefulness of policy relevant and comparable health information. The Joint Action for ECHIM contributes directly to the 2008–2013 Health Strategy of the European Commission [17].

DG SANCO has improved dissemination of ECHI indicators at the EU level by developing the HEIDI tool [12]. Another positive development is the Eurostat regulation on statistics on public health and health and safety at work [18], including the implementing regulation on EHIS [19], which refer to the ECHI shortlist. These will support the improved comparability of data across countries. Furthermore, increasing cooperation between the European Commission, WHO Regional Office for Europe and OECD, for example in the field of health expenditures statistics, is resulting in better data quality and comparability and less administrative burden for the Member States. Finally, the Member States have been involved in all the development steps of ECHI/ECHIM, and they increasingly use the ECHI shortlist for their own health information strategies. Nevertheless, despite years of work, substantial efforts are still needed at both Commission and national level to build-up and maintain a sustainable, high quality and easy-access evidence base for policy makers and other professionals. An important reason for this is that EU activities related to the development of elements of a health information system (e.g. indicators, standards for data collection, reporting mechanisms) are usually funded on an ad hoc project basis, resulting in a scattered and unsustainable situation.

Therefore it may not come as a surprise that after the ending of the Joint Action for ECHIM in June 2012, no follow up structure was put in place by the Commission. The Commission is performing a formal evaluation of the use and usefulness of ECHI in/for the Member States in 2013 and thereafter a decision about ECHI’s future will be taken. The success and future of ECHI, however, depends on the ability of a central EU organization to organise and implement the collection and use of ECHI data at EU level in health monitoring and reporting. A health information system like ECHI needs constant maintenance, e.g. in the form of metadata compilation and indicator improvement. In addition, the countries need to be able to provide required data in a timely way and with sufficient quality, preferably through international data collection systems. The Member States have a major role to play, since they have to implement the ECHI Indicators in practice.

The Joint Action faced several obstacles that call for improvements. Most importantly, a stronger leadership at EU level and enhanced funding, dedicated personnel, and commitment at national level are needed in order to speed up the action. In the future, the logical and viable perspective would be to integrate the work on ECHI‒defined data both with the collection of national health data and with the delivery of data to other international databases, such as those of Eurostat, WHO and OECD. Such a solution would be a coherent investment of resources, aimed at constantly improving the availability and cross‒national comparability of health data.

Finally, health information and knowledge should be emphasized in the forthcoming Health for Growth Programme for 2014–2020. The Joint Action for ECHIM has prepared good documentation and methods to support the national and international work on health indicators, health monitoring and health reporting.

## Conclusions

The ECHI Indicators were developed as the core of a European health information and reporting system, and have proven their usefulness and added value in practice. Sustained efforts at EU and national level are needed to keep the system functional and up to date.

## Abbreviations

DG SANCO: European commission´s directorate general for health & consumers; DPS: Data presentation system; ECDC: European centre of disease prevention and control; ECHI: European community health indicators; ECHIM: European community health indicators and monitoring; EHIS: European health interview survey; EFTA: European free trade association; EMCDDA: European monitoring centre for drugs and drug addiction; EU: European union; Eurostat: Statistical office of the European union; HEIDI: Health in Europe: information and data interface; OECD: Organisation for Economic Co-operation and Development; WHO: World Health Organisation.

## Competing interests

The authors declared that they have no competing interest.

## Authors’ contributions

All authors participated in the design of the study and data preparation. MV, MG and KK drafted the paper with the help of AT-N and A-PS. All authors reviewed and commented on the manuscript before submission and approved the final version.
